# Food digital marketing on social media: trends and strategies of Brazil’s leading meal delivery app

**DOI:** 10.3389/fnut.2025.1620348

**Published:** 2025-07-01

**Authors:** Paloma Aparecida Anastacio Barros, Juliana de Paula Matos, Michele Bittencourt Rodrigues, Renata Júlia da Costa, Paula Martins Horta

**Affiliations:** Programa de Pós-graduação em Nutrição e Saúde – Universidade Federal de Minas Gerais, Belo Horizonte, Brazil

**Keywords:** social media, marketing, mobile applications, processed food, advertisement

## Abstract

**Objective:**

To analyze digital marketing trends of Brazil’s leading meal delivery application (MDA) company on Facebook (FB) and Instagram (IG) from 2011 to 2022.

**Methods:**

This exploratory, longitudinal, and mixed-methods study examined a 10% sample of all posts published by this company during the study period. Posts were analyzed in terms of food categories, media and connectivity features, and advertising themes.

**Results:**

The company predominantly promoted unhealthy foods, frequently employing persuasive digital marketing strategies. While this pattern was consistent across both platforms, IG posts were more visually engaging and interactive, making greater use of brand elements, hashtags, conversations, emoticons, user interaction, and company tagging (all *p* < 0.001). In contrast, FB posts more commonly included external links. Twelve distinct advertising themes were identified. They reflected a broad and diverse range of marketing strategies, encompassing brand promotion, user engagement, contextual appeals, technological features, social responsibility, and other themes. IG posts more frequently featured themes such as *entertainment and social interaction*, *consumption stimulus*, *application differentials*, *public figure endorsements*, and *social and corporate responsibility* (all *p* < 0.05). Conversely, the theme of *communication and news* was more prevalent on FB. Longitudinal analysis revealed that the company adapted its content over time, refining its use of media tools and thematic strategies in response to evolving digital marketing.

**Conclusion:**

The company primarily advertised unhealthy foods on both social media, leveraging persuasive techniques and marketing strategies. These practices may hinder public health efforts to reduce the consumption of unhealthy foods.

## 1 Introduction

Meal delivery apps (MDAs) are online platforms that manage meal ordering by connecting food establishments with consumers (1). Accessible via internet-enabled devices, these apps allow users to browse menus, read reviews, place orders, and receive meals conveniently anywhere (1). MDAs play a significant role in enhancing food accessibility and availability, thereby influencing population dietary patterns (1, 2).

The use of MDAs has grown substantially worldwide, particularly among younger populations attracted by convenience, practicality, and time efficiency (1, 3, 4). By 2024, global use of online food delivery services had surpassed 2.5 billion users (5), with Brazil accounting for approximately 80 million users (6).

Previous studies have documented the widespread availability of meals with low nutritional quality and high energy density on these platforms across diverse global settings (7–16). This trend has raised concerns among public health authorities (1), as frequent consumption of ultra-processed foods has been linked to overweight, obesity, and chronic noncommunicable diseases (17, 18).

To promote their services, expand market reach, and retain customers, MDA companies have heavily invested in digital marketing strategies, particularly through social media (1). Social media platforms are defined as technological ecosystems that connect individuals, businesses, organizations, and institutions (19). According to media richness theory, organizations and consumers utilize multiple media channels to communicate (20). These platforms enable companies to reach large audiences, foster interaction and engagement, and advertise products and services effectively (21, 22). Moreover, digital channels allow for high-volume information dissemination and reduce the perceived cost of information search. They also facilitate knowledge exchange and provide rapid, tailored feedback, thereby influencing consumer decision-making (20).

In Brazil, social media is among the most widely used online activities, especially among younger individuals (23). Facebook (FB) and Instagram (IG) are two of the most prominent platforms, with approximately 154 million and 113 million users, respectively (23). These platforms serve distinct functions: FB emphasizes user profiles and social connections, whereas IG focuses on visual content dissemination, with user interactions primarily driven by shared interests and content affinity (22, 24). These differences have prompted companies to develop platform-specific marketing strategies (22).

Although some studies have explored the digital marketing strategies of MDA companies, only three have monitored their social media activities, primarily in the context of the COVID-19 pandemic (25, 26, 27). In Australia, the United Kingdom, and the United States, IG posts by MDA companies included links, images, brand elements, and content related to pandemic response efforts, including corporate social responsibility actions (25). In Brazil, researchers observed changes in the advertising strategies of three major MDA companies on IG when comparing posts from the early months of the pandemic to the preceding six months (26). Specifically, there was a decline in food imagery particularly of ultra-processed products and an increased focus on messages related to corporate responsibility and public health (26). Another Brazilian study analyzed 137 posts from three MDA companies across FB, IG, and YouTube during the pandemic, finding that approximately 58% featured unhealthy foods, with content centered on branding, COVID-19 information, and corporate social responsibility (27).

The present study aims to build on this body of research by conducting the first comprehensive analysis of digital marketing trends by Brazil’s leading MDA company on social media between 2011 and 2022. The findings will enhance understanding of how this company utilizes different platforms and may inform regulatory discussions regarding the promotion of unhealthy foods in digital environments.

## 2 Methods

### 2.1 Outline of the study and company profile

This was an exploratory, longitudinal, and mixed-methods study. Exploratory studies have been used to assess digital marketing strategies on food company social media platforms (28), particularly given the dynamic nature and limited prior research in this field. The mixed-methods approach (qualitative and quantitative) permits a more comprehensive assessment of advertising content. Content analysis, previously employed in other studies on food marketing in social media (29, 30), allows for the identification of specific strategies, platform-exclusive features, and company behavior.

The company analyzed was founded in 2011 in Brazil and has since become the national leader in the online food delivery sector, operating in more than 1,700 cities (31). As of January 2023, it reported 65 million monthly orders, over 300,000 registered restaurants, and more than 1.5 million monthly app downloads (31).

### 2.2 Data collection and variable organization

The dataset included all posts published by the company on FB (*n* = 3,153) and IG (*n* = 1,838) in Brazil. Data were collected using Fanpage Karma, an automated tool that retrieved each post and its engagement metrics (likes, comments, and shares). The tool exported the data in Excel format. Posts were categorized by publication date and engagement metrics. The number of followers on each platform was also recorded in December 2022.

To ensure feasibility, a 10% stratified random sample was drawn. Strata were defined by year and month, ensuring proportional representation across the study period. This subsample (*n* = 325 FB; *n* = 187 IG) was analyzed to identify food images, based on predefined categories: traditional meals (e.g., rice, beans, meat, salad), fruits, greens, and vegetables (FGV) (e.g., fruit salad, vegetable soup), healthy snacks (e.g., fruit pancakes), healthy beverages (e.g., fresh orange juice), unhealthy snacks (e.g., hamburgers, pizza), alcoholic beverages (e.g., beer), ultra-processed beverages (e.g., soft drinks) and other foods not classified above. These categories were adapted from prior research on the food environment in MDAs in Brazil and are consistent with the Brazilian Dietary Guidelines and the NOVA classification system (8, 13, 14, 32) (Table 1).

**Table 1:** Criteria used to classify food types, meals, and media/connectivity elements in posts by the leading meal delivery app company on Facebook and Instagram in Brazil.

Variable	Description
Type of food	
Traditional meals dishes	The typical Brazilian meals (e.g., preparations with rice, beans, meat, and salad) or international cuisine (e.g., oriental dishes and pasta). These dishes are predominantly made with unprocessed and minimally processed foods.
Fruits, greens, and vegetables (FGV)	Preparations predominantly comprised fruits, vegetables, and greens (e.g., banana, fruit salad vegetable soup).
Healthy snacks	Sweet or savory snacks, predominantly made with unprocessed or minimally processed ingredients (e.g., fruit pancakes, couscous, corn cake, and sandwiches made with bread, meat, egg or cheese, and vegetables).
Healthy beverages	Bottled mineral drinking water or beverages made with fruits or vegetables (e.g., freshly squeezed orange juice and pineapple juice).
Unhealthy snacks	Sweet or savory snacks, made predominantly with ultra-processed ingredients (e.g., hamburgers, hot dogs, pizza, snacks, French fries, fried chicken, chocolates, ice cream, açaí, milkshake, and other goodies such as churros, pies, brownies, brigadeiro—a type of Brazilian chocolate confectionery).
Alcoholic beverages	Alcoholic beverage (e.g., beer and wine).
Ultra-processed beverages	Soft drinks, industrialized juices, and energy drinks.
Others	Other foods and meals not included in the above categories (e.g., egg, milk, and oil).
Media and connectivity resources	
Post type	Classification of the post in photo or image; video and GIFs/Boomerang (photos and moving images); written text only.
Link	Presence of a link to an external page or additional content. e.g.: Link in the post directing to other pages, such as the company’s app.
User interaction	This involves tagging other user profiles or sharing user-generated content, such as photos and videos, which are posted in conjunction with the brand on the timeline/feed.
Food company tagging	Presence of tagging profiles of food companies and restaurants (e.g., Coca-Cola, McDonald’s).
Hashtags	Presence of the symbol # followed by a keyword to mark a message and facilitate a search (e.g., “#love,” “#ifood,” #ifoodsalva” and “delivery”).
Emoticons	Presence of emoticons (i.e., graphic resources that represent reactions or food/objects).
Brand elements	Presence of brand characterization elements such as logos, brand colors, fonts, trademarks, or slogans.
Engagement	Presence of a stimulus to an online action by the user (e.g., the brand asks the user to like, comment or share content).
Conversations	Presence of brand responses to users in post comments.

*Note*: A post can contain one or more types of food and meals or media and connectivity resources.

The same subsample was analyzed for media and connectivity elements, including: type of post, external links, user interaction, company tagging, hashtags, emoticons, brand elements, engagement, and conversation prompts. A structured protocol was developed based on prior literature on digital food marketing practices (13–14, 25, 29–30, 33–34) (Table 1). Due to the subjectivity in identifying some variables, the full protocol was applied by the lead researcher. A second researcher independently coded 10% of the subsample, resulting in a 97% inter-rater agreement rate, ensuring reliability and consistency.

To analyze visual and textual content, a thematic analysis was conducted, following Braun and Clarke’s method (35). This qualitative approach enables the identification, analysis, interpretation, and reporting of explicit and implicit themes. It is valued for its flexibility, accessibility, and ability to synthesize large datasets (35).

Four trained researchers conducted the analysis on a 10% post subsample (separately for each platform) through seven steps:

- pre-analysis: two researchers initiated the process by conducting an initial analysis and took notes of the principal themes (messages and appeals) present in each post.
- generating initial codes: carried out by the main researcher, who systematically coded the pre-analysis of the annotated data and categorized the relevant information for each code. This step resulted in 47 initial codes for FB and 38 for IG;
- searching for categories: the main researcher organized the codes into potential categories, including all relevant data for each potential category. This step resulted in 18 categories for FB and 16 for IG;
- review of categories: after reviewing the coded data and categories, a thematic ‘map’ was generated for analysis and discussed by all researchers. This step refined the categories and totaled a set of 12 categories for both FB and IG;
- treatment of categories: two researchers carried out a general analysis of all categories to enhance their specificity and establish precise definitions and labels. Subsequently, these categories were discussed among a group of three researchers.
- interpretation of results: two researchers independently analyzed the posts according to the categories found in the previous steps;
- analysis of the consistency of interpretation results: all results were compared (93.8% agreement rate) and disagreements were verified by a third researcher.

### 2.3 Data analysis

In the full sample (*n* = 3,153 FB; *n* = 1,838 IG), the total number of likes, comments, and shares per year was aggregated and visualized through line graphs for each platform.

In the subsample (*n* = 325 FB; *n* = 187 IG), the frequency of food categories, media/connectivity features, and thematic categories was calculated for each year and platform. Differences between platforms were tested using Pearson’s Chi-square test, with a 5% significance level. Analyses were conducted using Stata software, version 14.

For the same subsample, longitudinal trends in the presence of food categories, media and connectivity elements, and thematic content were described using trendline graphs by year and platform, based on relative frequencies.

## 3 Results

**Figure 1:**
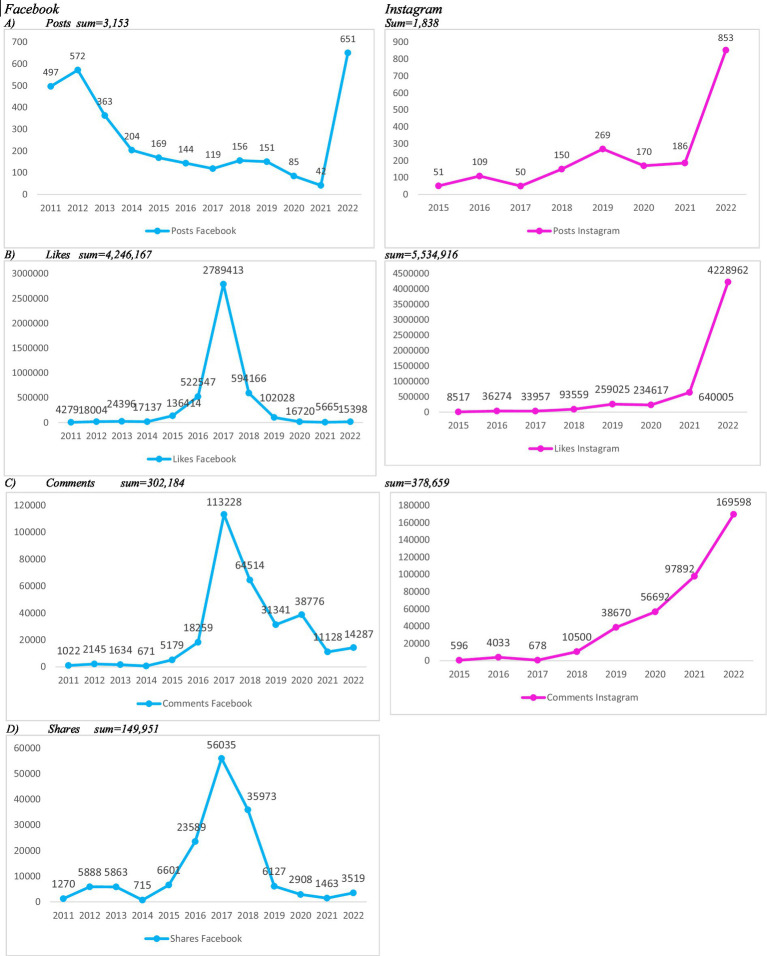
Number of posts by the main meal delivery app company on Facebook and Instagram in Brazil and number of interactions received, 2011–2022.

As of December 2022, the company had 2,196,422 followers on FB and 2,162,738 on IG (data not shown in the figure). The company’s FB page, created in 2011, reached its peak volume of posts in 2022 (*n* = 651). The IG account, launched in 2015, also registered its highest posting activity in 2022, with 853 posts (Figure 1A).

Throughout the study period, FB showed a general decline in posting frequency, despite a temporary surge in 2022. In contrast, IG exhibited a steady increase in the number of posts over time (Figure 1A). Cumulatively, FB posts received 4,246,167 likes, while IG posts garnered 5,534,916 likes (Figure 1B). Similarly, total comments amounted to 302,184 on FB and 378,659 on IG (Figure 1C). Data on shares were available only for FB, totaling 149,951 (Figure 1D).

The highest levels of engagement on FB occurred in 2017, with 2,789,413 likes, 113,228 comments, and 56,035 shares. On IG, peak engagement was observed in 2022, with 4,228,962 likes and 169,598 comments. Overall, FB interaction data between 2011 and 2022 did not display a clear upward or downward trend, except for the notable spike in 2017. Conversely, IG showed a consistent upward trajectory in both publication volume and user engagement. These patterns are illustrated in Figures 1A–D.

In the analyzed subsample, food images appeared in 68.6% of FB posts and 67.9% of IG posts, with no statistically significant difference between platforms (*p* = 0.870) (data not shown in the table). Unhealthy snacks were the most frequently featured food category, appearing in 60.1% of FB posts and 67.7% of IG posts (*p* = 0.156). Traditional meals were more frequently depicted on FB (43.9%) than on IG (33.1%) (*p* = 0.046). In contrast, ultra-processed beverages appeared more often on IG (11.8%) than on FB (4.5%) (*p* = 0.010). On both platforms, posts featuring FGV, healthy snacks, or healthy beverages accounted for less than 11% of the content (Table 2).

**Table 2:** Types of food, meals, and media/connectivity resources present in posts by the leading meal delivery app company on Facebook and Instagram in Brazil, 2011–2022.

Variables	Facebook (*n* = 325)		Instagram (*n* = 187)		*p*
Types of food or meals	N	%	N	%	
Unhealthy snacks	134	60.1	86	67.7	0.156
Traditional meals	98	43.9	42	33.1	**0.046**
Alcoholic beverages	21	9.4	15	11.8	0.478
Fruits, greens, and vegetables (FGV)	19	8.5	14	11.0	0.441
Healthy snacks	14	6.3	7	5.5	0.772
Ultra-processed beverages	10	4.5	15	11.8	**0.010**
Healthy beverages	8	3.6	3	2.3	0.528
Others	6	2.3	6	4.7	0.315
Media and connectivity resources					
Post type					
Photo/Image	243	74.7	98	52.4	**<0.001**
Vídeo, GIF’s or boomerang	62	19.1	89	47.6	
Text without visuals	20	6.1	0	0	
Brand elements	245	75.4	167	89.3	**<0.001**
Link	232	71.4	5	2.6	**<0.001**
Hashtags	91	28.0	148	79.1	**<0.001**
Emoticons	90	27.7	112	59.9	**<0.001**
Engagement	84	25.8	58	31.0	0.208
Conversations	68	20.9	115	61.5	**<0.001**
User interaction	61	18.7	90	48.1	**<0.001**
Food company tagging	16	4.9	31	16.6	**<0.001**

Bolded values indicate statistically significant results based on Pearson’s chi-square test with *p* < 0.05.

**Figure 2:**
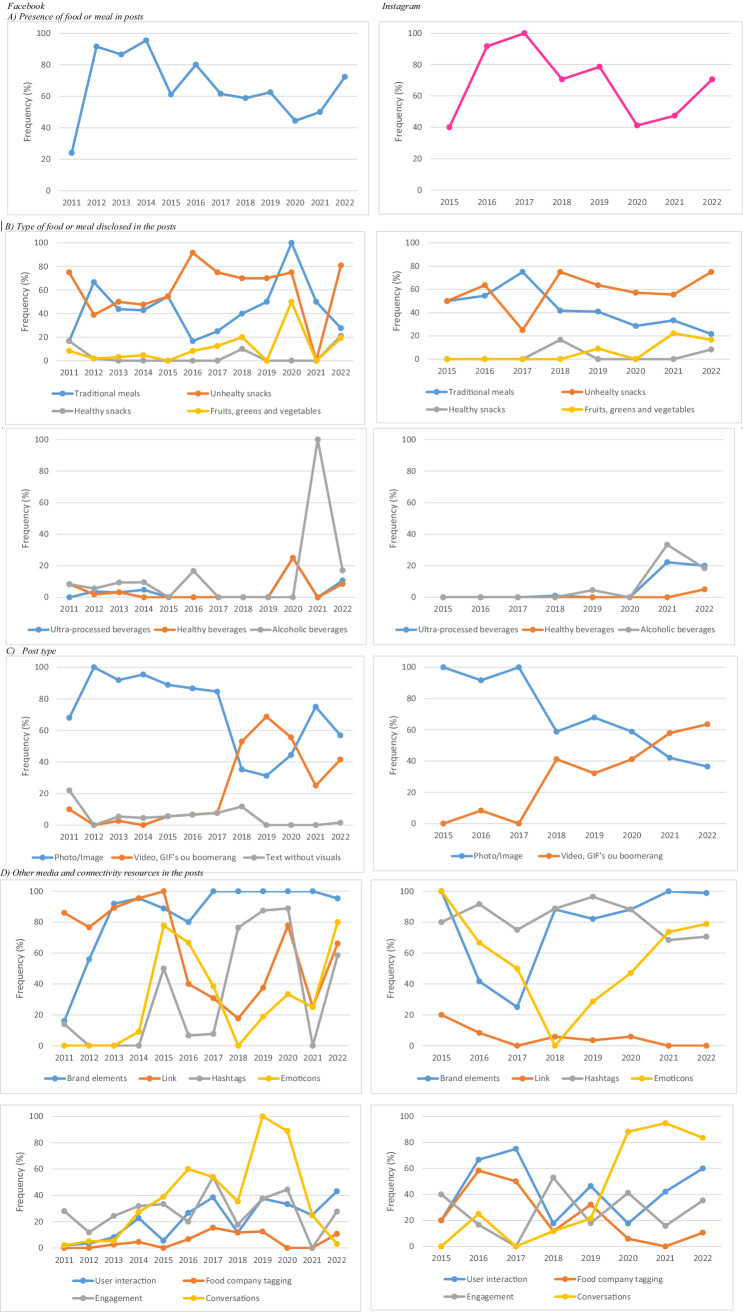
Longitudinal analysis of food types and media/connectivity resources in posts by the leading meal delivery app company on Facebook and Instagram in Brazil, 2011–2022.

Longitudinal analysis revealed a general decline in the use of food images on FB posts from 2014 onward and on IG from 2017 onward. However, both platforms showed an upward trend in such images starting in 2020 (Figure 2A). Regarding specific food categories, no clear pattern emerged for traditional meals on FB, while IG posts showed a downward trend over time (Figure 2B). The frequency of posts featuring alcoholic beverages remained relatively stable across both platforms, with a notable increase in 2021 (Figure 2B).

Analysis of media and connectivity resources revealed significant differences between social media platforms (*p* < 0.001). IG posts more frequently featured dynamic visual content (such as videos, GIFs, or boomerangs) compared to FB (47.6% vs. 19.1%). In contrast, FB posts relied predominantly on static images (74.7% vs. 52.4% on IG), with text-only posts observed exclusively on this platform (6.1%). FB also exhibited a higher prevalence of links (71.4% vs. 2.6% on IG; *p* < 0.001). Conversely, IG posts more often incorporated brand elements (89.3% vs. 75.4%), hashtags (79.1% vs. 28.0%), conversational content (61.5% vs. 20.9%), and emoticons (59.9% vs. 27.7%) (all *p* < 0.001). Interaction features were also more common on IG, including user engagement (48.1% vs. 18.7%) and tagging of food companies (16.6% vs. 4.9%) (*p* < 0.001; Table 2).

Regarding trends in media and connectivity resources over time, dynamic formats such as videos, GIFs, and boomerangs increased on both platforms from 2017 onwards, while photo/image posts declined on IG (Figure 2C). The use of links fluctuated on FB, whereas it remained consistently low on IG (Figure 2D). Hashtag use was more frequent and consistent on IG throughout the period, while on FB it followed a less regular pattern (Figure 2D). Emoticon use declined on both platforms between 2015 and 2018, followed by an increase in 2019. Conversations became more frequent on IG posts from 2017 onward, while their presence declined on FB after 2019. In contrast, company tagging decreased on IG over time and remained stable on FB (Figure 2D). Examples of posts illustrating these features are provided in Supplementary Material 1.

Content analysis identified 12 thematic categories, as detailed in Table 3. The most prevalent themes on both platforms were *brand institutional characteristics* (69.2% on FB vs. 75.9% on IG; *p* = 0.105) and *sensory* appeals (54.7% on FB vs. 54.0% on IG; *p* = 0.868). The former encompassed posts highlighting brand identity through slogans, brand colors, or corporate initiatives, such as the campaigns “Living on Delivery” and “One Year of Free iFood.” The *sensory* category included content emphasizing food attributes like texture and visual appeal, often employing vivid language and imagery designed to trigger cravings.

**Table 3:** Description of thematic categories in posts by the leading meal delivery app company on Facebook and Instagram in Brazil, 2011–2022.

Categories	Description	Facebook (*n* = 325)		Instagram (*n* = 187)		*p*
		N	%	N	%	
Institutional characteristics of the brand	The posts showed the brand’s characteristics, such as slogan, brand colors, and the company’s institutional values. Furthermore, they highlighted the criteria for choosing partner restaurants and the brand’s new campaigns, such as “Desafio Viver de Delivery” (In English: Challenge – Living on delivery) and “1 ano de Ifood grátis” (In English: One year of free Ifood)	225	69.2	142	75.9	0.105
Sensory	Posts that explored the sensory characteristics of food, through visual and text resources reinforced, for example, that the food was juicy and mouthwatering	178	54.7	101	54.0	0.868
Entertainment and social interaction	The posts covered promotions of concerts, parties, TV series launches, sports events, and video game tournaments. Additionally, they could also involve interactions or engagements between the company, users, and partners, as well as the use of humor and emotional appeals to personal relationships	89	27.4	78	41.7	0.001
Economic benefit	The posts referred to promotions, discounts, combos, free deliveries, use of giveaways, and contests. Furthermore, they highlighted economic affordability through phrases like “fits any budget” and “fair pricing,” as well as the availability of various payment methods (e.g., payment with meal vouchers, Ifood card)	86	26.4	62	33.1	0.108
Consumption stimulus (in specific situations and contexts)	The posts encouraged consumption through depictions of food and various eating situations and contexts, such as *(i)* mindless eating (e.g., eating while using digital devices or watching TV); *(ii)* overeating (e.g., large portions); *(iii)* eating as a reward (e.g., after a workday, one deserves a delicious snack); *(iv)* references to specific contexts, like sporting events (e.g., soccer day) or seasons of the year (e.g., “enjoy the sunny day by the pool with Ifood”); and *(v)* associations with the eating context, like eating with children, family members, or friends	86	26.4	68	36.3	0.019
Practicality and convenience	The posts referred to convenience and ease when placing an order, highlighting aspects like time-saving, not needing to cook, avoiding traffic/queues, and substituting lunch with quick snacks	84	25.8	57	30.5	0.258
Application differentials	The posts reinforced the app’s differentials features, conveniences, and benefits for placing orders, as well as the company’s latest releases and new functionalities, such as online grocery shopping, pharmacy, and pet shop options	64	19.7	72	38.5	<0.001
Food culture and sophistication	Posts highlighting refined cuisine from diverse cultures and regions, while featuring gourmet dishes through images of harmonized pairings and elegant dining establishments	61	18.7	40	21.4	0.473
Endorsement of public figures	The posts featured publicly recognizable figures and/or celebrities offering tips and suggestions for orders, sharing lists of their favorite restaurants, and providing recommendations for home-based activities	39	12.0	66	35.3	<0.001
Thematic campaigns	The posts elucidated how users could utilize the application during commemorative occasions (e.g., Father’s Day or Valentine’s Day) and specific events (e.g., Saint John’s Day or World Cup)	38	11.7	36	19.2	0.019
Communication and news	Posts with the dissemination of information from alternative communication platforms, such as external websites (e.g., Ifood News and Ifood Blog), include content concerning dietary choices, nutritional merits of foods, the app’s most frequently requested items, the company’s narrative of achievements, delivery service expansion, digital technologies, and the utilization of electronic devices	27	8.3	7	3.7	0.046
Social and corporate responsibility	The posts highlighted the brand’s social and corporate responsibility actions, including the dissemination of initiatives directed toward partners, delivery personnel, and restaurants. This includes support for vulnerable populations, the incorporation of a “donation” section within the app, as well as the company’s ventures and projects dedicated to environmental sustainability. During the COVID-19 pandemic, posts accentuated protective measures such as contactless delivery, alongside investments undertaken by the company, including the establishment of a solidarity fund	6	1.8	12	6.4	0.007

Several themes appeared significantly more frequently on IG than on FB, reflecting distinct communication strategies. One of the most prominent was *entertainment and social interaction* (41.7% on IG vs. 27.4% on FB; *p* = 0.001), encompassing posts that promoted concerts, parties, TV series, sports, and events. These often featured interactions between the company, users, and partners, employing humor and emotional appeals related to personal relationships. The theme *consumption stimulus in specific situations and contexts* (36.3% on IG vs. 26.4% on FB; *p* = 0.019) included posts portraying various eating scenarios: (i) mindless eating (e.g., while watching TV); (ii) overeating (e.g., large portions); (iii) eating as a reward (e.g., after work); (iv) references to specific events (e.g., soccer matches); and (v) social eating contexts, such as meals with family or friends. The category *application differentials* (38.5% on IG vs. 19.7% on FB; *p* < 0.0001) highlighted app-related benefits (ease, speed, and variety) as well as new services, including grocery delivery, pharmacy access, and pet-related products. *Endorsement of public figures* (35.3% on IG vs. 12.0% on FB; *p* < 0.0001) was notably more frequent on IG, where celebrities and influencers promoted meals, recommended restaurants, or suggested at-home activities. These endorsements aimed to foster trust and emotional connection through familiar personalities.

The themes *themed campaigns* (19.2% on IG vs. 11.7% on FB; *p* = 0.019) and *social and corporate responsibility* (6.4% on IG vs. 1.8% on FB; *p* = 0.007) were also more prevalent IG. *Themed campaigns* referred to posts aligned with commemorative dates (e.g., Valentine’s Day) or major events (e.g., the World Cup). Posts under *social and corporate responsibility*, particularly during the COVID-19 pandemic, emphasized protective measures (e.g., contactless delivery) and highlighted the company’s involvement in solidarity initiatives, sustainability actions, support for delivery workers, and food donations. Other themes, although not statistically different between platforms, were still relevant. *Economic benefit* (33.1% on IG vs. 26.4% on FB; *p* = 0.108) included posts offering discounts, meal combos, and free delivery. *Practicality and convenience* focused on the app’s ease of use and time-saving advantages, frequently positioning delivery as an alternative to home cooking. *Food culture and sophistication* (21.4% on IG vs. 18.7% on FB; *p* = 0.473) encompassed content promoting gourmet or refined dishes, often paired with curated combinations and references to regional or international cuisines.

Only the category *communication and news* appeared more frequently on FB than on IG (8.3% vs. 3.7%; *p* = 0.046). Posts in this category often redirected users to external sources, such as the company’s blog, where content included nutritional information, benefits of specific foods (e.g., ice cream), and rankings of the most frequently ordered items on the app (Table 3). Examples of each thematic category are provided in Supplementary Material 2.

**Figure 3:**
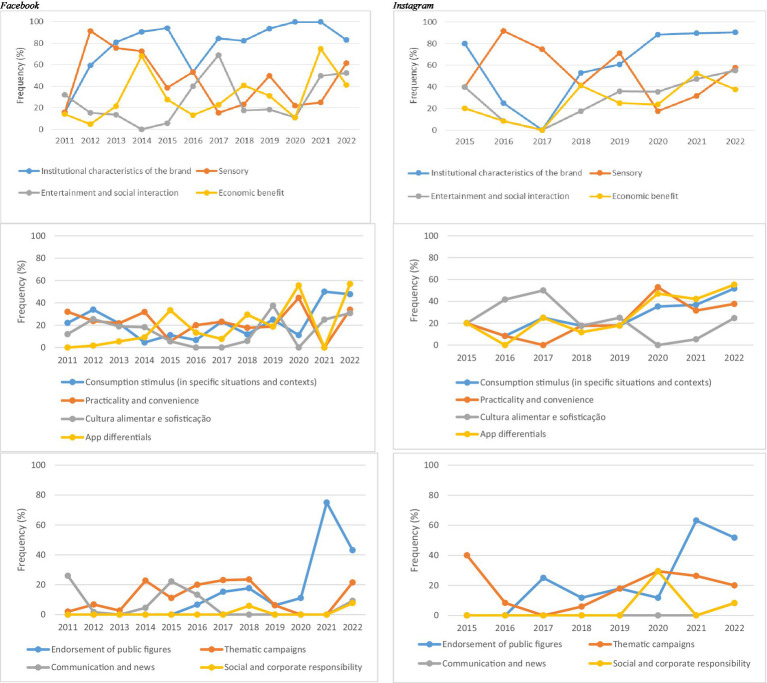
Longitudinal analysis of thematic categories in posts by the leading meal delivery app company on Facebook and Instagram in Brazil, 2011–2022.

An analysis of thematic categories over time reveals distinct trends across platforms. On FB, *institutional characteristics of the brand* showed a generally upward trajectory, except for a marked decline in 2016. On IG, this theme initially declined but grew steadily from 2017 to 2020, maintaining high frequency through 2022. The category *entertainment and social interaction* followed a defined pattern on IG, with a decline between 2015 and 2017, followed by consistent growth—distinct from the trend observed on FB. The themes *consumption stimulus in specific situations and contexts*, *practicality and convenience*, and *application differentials* remained relatively stable on FB but showed increasing frequency on IG over time. The category *endorsement of public figures* was introduced in 2015 on FB and in 2016 on IG. *Social and corporate responsibility* was the most recent theme to emerge, first appearing on FB in 2018 and gaining prominence on IG from 2020 onward. Finally, *communication and news* remained stable on FB and was used on IG for the first time only in 2022 (Figure 3).

## 4 Discussion

This study described, for the first time, the digital marketing content of the largest MDA company in Brazil on FB and IG, covering the period from 2011 to 2022. The findings show that the company consistently used social media platforms to promote its services through posts predominantly featuring unhealthy foods, marked by high interactivity and persuasive appeal.

The intensive use of digital marketing strategies by unhealthy food companies has raised significant public health concerns, as widespread exposure to such content may negatively affect health (28, 36). Among young people (12 to 30 years old), the most active users of digital media, exposure to this type of marketing increases the acceptability of unhealthy products and reduces the perceived risks of their consumption (21). Although evidence regarding adults remains limited (36), food companies have targeted this group with substantial volumes of digital marketing in several countries (37).

The predominance of posts promoting unhealthy products such as sweetened drinks, ice cream, and candy contrasts sharply with the limited promotion of FGV, healthy snacks, and beverages. This underscores the role of social media as a powerful tool for promoting foods offered by MDA establishments. Two studies, one cross-sectional (14) and one longitudinal (8), reported that over 50% of menu items available on MDAs in a Brazilian capital were unhealthy, including ultra-processed foods (8, 14).

Over the years, advertising patterns have shifted. Since 2014 on FB and 2017 on IG, there was a decline in food image posts. The COVID-19 pandemic likely influenced the company to realign its advertising strategy, incorporating more health-related content alongside the promotion of MDA offerings (38).

Another notable shift was the increased presence of alcoholic beverages in the company’s posts from 2020 onward. This trend coincides with the onset of the COVID-19 pandemic and a marked rise in alcohol consumption among the Brazilian population. Data from the Surveillance of Risk and Protective Factors for Chronic Diseases by Telephone Survey (VIGITEL) indicate that 2020 recorded the highest proportion of Brazilian adults reporting excessive alcohol consumption (20.9%) (39). A recent scoping review has highlighted concerns about the availability of alcoholic beverages via MDAs, especially given the insufficient enforcement of age restrictions (40). While conventional food and beverage establishments in Brazil explicitly prohibit the sale of alcohol to individuals under 18 years of age, the platform studied facilitates this practice. Customers purchasing alcoholic beverages on the platform are required only to confirm their age (“Yes, I am over 18 years of age”) before completing the transaction.

Comparing the two platforms, IG promoted more ultra-processed beverages and fewer traditional meals than FB. This aligns with user demographics: in 2020, 35% of Brazilian IG users were aged 18–29, while only 15% were 50 or older (41). On FB, these figures were 30 and 18%, respectively (41). In Brazil, younger individuals tend to have less healthy diets (42). Thus, the food profile promoted by the company on IG may be unintentionally reinforcing unhealthy eating habits among young people.

Furthermore, the company’s choice of communication platform is shaped not only by technical features but also by user attitudes, prior experience, platform knowledge, and social context (20, 43–44). These factors may help explain the company’s distinct engagement strategies across platforms. In terms of media and connectivity resources, the use of brand elements stood out on both platforms. This is a common strategy in food marketing on social media and is also widespread among MDA companies (45, 25–27). Consistent brand elements support consumer recognition and help build long-term relationships between companies and audiences. According to Maity et al. brand elements act as symbolic representations of the company and influence both platform choice and communication style (43).

Visually rich posts such as images, videos, GIFs, and boomerangs, were more common than purely textual content, especially on IG and in recent years. IG’s tools (e.g., transitions, audio, visual effects) make it easier to create short, engaging videos, which are well-suited to capturing attention through dynamic content (46, 47). According to Media Richness Theory (20, 43–44), IG qualifies as a “rich medium”: it enables immediate feedback, combines multiple cues (video, audio, visuals), and conveys emotion effectively. This aligns with the preferences of younger users, who are drawn to fast-paced, visually stimulating content. In contrast, FB may be considered a “leaner” medium, less stimulating but more conducive to memory retention through linear, text-based interaction. Based on this, richer media may drive short-term engagement with minimal cognitive effort, while leaner media may enhance long-term brand recall. This suggests platform-specific strategies: on IG, brands may prioritize volume, interactivity, and visual impact; on FB, they may focus on message clarity and reinforcing brand identity (20, 43–44).

The company also used features designed for digital interaction, such as hashtags, emoticons, user tagging, engagement prompts, and conversations, especially on IG. Hashtags increase visibility beyond followers; emoticons add emotion or context; tagging supports the spread of user-generated content without requiring explicit advertising disclosure. Engagement prompts (like calls for likes, shares, or comments) amplify message reach. Conversations offer a channel for the company to hear user experiences and feedback. Tagging other food companies in a post fosters collaboration and broadens the ad’s target audience. These tools highlight IG’s strength in enabling interactive, emotionally resonant, and high-reach marketing campaigns (48). In contrast, FB’s more static environment may be better suited for strategic messaging aimed at an older, more passive audience. Once again, Media Richness Theory helps explain these choices: platforms are not neutral but are differently suited to particular types of communication and audiences (20, 43).

The thematic analysis revealed 12 categories. Many reflect common dimensions of food marketing across different media types. For example, studies on television advertising often track sensory appeals, consumption stimuli—such as usage suggestions or targeted recommendations (e.g., for children)—and economic benefits, including contests, price discounts, or loyalty programs (49). Other similarities between traditional and digital media include the use of public figure endorsements and thematic campaigns tied to events or commemorative dates (49).

Other themes identified were specific to the digital environment. Notably, *entertainment and social interaction* and *communication and news* reflect the interactive nature of social media content. Prior research has shown that this includes the use of media tools, such as apps, games, links, hashtags, and emoticons, as well as strategies to promote user interaction, like liking, commenting, sharing, or downloading content (45).

Our study also revealed themes characteristic of MDA services. The theme *practicality and convenience* aligns with contemporary lifestyles marked by long working hours and the pursuit of efficiency, particularly in domestic tasks (1). The appeal of meal delivery without leaving home and the ease of kitchen organization help explain the popularity of these posts (1). The theme *application differentials* refers to the promotion of new app features and user benefits during the ordering process. These functionalities stem from major technological investments by MDAs, including artificial intelligence, and are personalized using user data—such as browsing history, order patterns, timing, location, and device used (1). The theme *food culture and sophistication* also emerged in the posts, reflecting the inclusion of “haute cuisine” restaurants in the platform, aimed at a higher-income (50).

Another recurring theme was *social and corporate responsibility*, often highlighted during the COVID-19 pandemic (51, 52). Initiatives included food donations to vulnerable populations and messages promoting environmental sustainability, such as financial support for protecting Brazilian biomes (31). While these actions address relevant social and environmental issues, they also serve as strategic marketing tools (53). They help build a positive brand image, shape consumer preferences (54), and may reduce the perceived harm of certain products—ultimately encouraging the consumption of items that pose potential health risks (28, 54).

Regarding thematic differences between platforms, we observed that the company used FB mainly for informative or news-oriented content, while IG featured a wider range of themes. Voorveld et al. highlight that consumer engagement and advertising on social media vary according to contextual factors and personal interactions within each platform (24). FB is structured around user profiles, supporting relationship-building and personalized messaging. IG, on the other hand, is centered on creative content and the communal sharing of topics of interest (22, 24).

Based on the findings of the study, two main perspectives emerge: (i) monitoring MDA marketing: our results highlight the need to standardize and improve monitoring methods for MDA marketing in the digital environment. This includes evaluating the types of foods advertised, using platform-specific tools, and adopting qualitative approaches to better understand marketing strategies. Such efforts must consider the unique characteristics of each social media platform. And (ii) advancing the regulatory agenda: currently, Brazil lacks legal restrictions on digital food marketing by MDAs. Given the nature of these marketing strategies and their role in promoting unhealthy eating habits, consumer protection laws and regulations that ensure healthy eating should be expanded to cover this area.

This study has some limitations. It analyzed only one MDA company; however, this company dominates the Brazilian market (31). Additionally, the analysis focused solely on official social media posts, excluding other communication channels and personalized advertising formats. These include artificial intelligence powered tools and algorithms that track consumer behavior to tailor content (1, 16).

Despite these limitations, the study has notable strengths. It is the first to examine an MDA company’s performance across two distinct social media platforms over a 12-year period. This extended timeframe allows for comparison of diverse advertising tactics based on each platform’s unique features. The mixed-methods approach, combining qualitative and quantitative analyses, enabled a comprehensive evaluation of food types, social media tools, and thematic content in the advertisements.

In sum, the findings provide insight into how this company skillfully uses social media to promote less healthy foods and beverages. It employs contemporary, dynamic, and highly persuasive marketing strategies that engage the audience directly, responding to their needs while adapting to shifting societal values and evolving social and economic dynamics. The use of social media by MDA platforms has the potential to reshape dietary patterns and reinforce the consumption of ultra-processed foods and alcoholic beverages. This convergence of factors may make these digital spaces harmful to public health. Therefore, rigorous measures, such as regulating these platforms and their marketing strategies are essential to foster healthier food environments.

## Data Availability

The raw data supporting the conclusions of this article will be made available by the authors, without undue reservation. Requests to access these datasets should be directed to the corresponding author.
