# Supplementary material for: Food digital marketing on social media: trends and strategies of Brazil’s leading meal delivery app
Source: Front Nutr. 2025 Jul 1;12:1620348. doi: 10.3389/fnut.2025.1620348 (PMC12265301; doi:10.3389/fnut.2025.1620348)

SUPPLEMENTARY MATERIAL 1 - Examples of media and connectivity resources present in the posts of the main meal delivery app company on Facebook and Instagram, in Brazil 2011-2022.

A) Representation of a post with photo/image

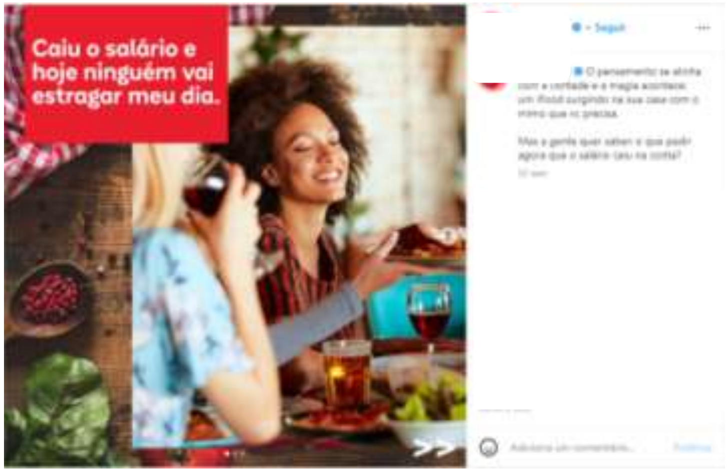

B) Representation of a vídeo post

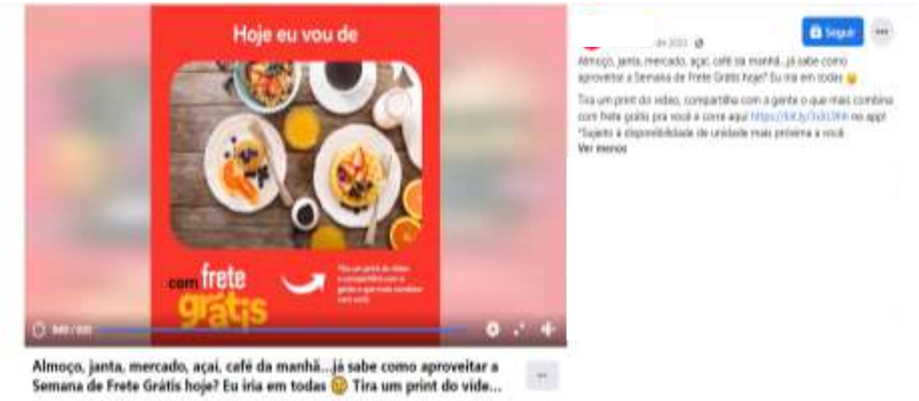

C) Text only post representation

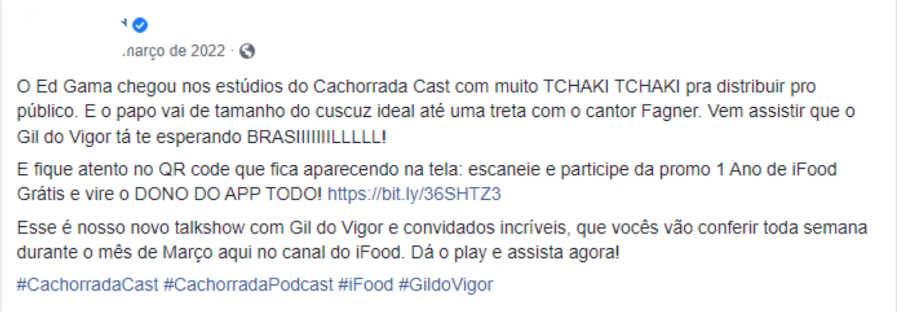

D) Presence of post brand elements (red color and logo), link (to acess the brand website)

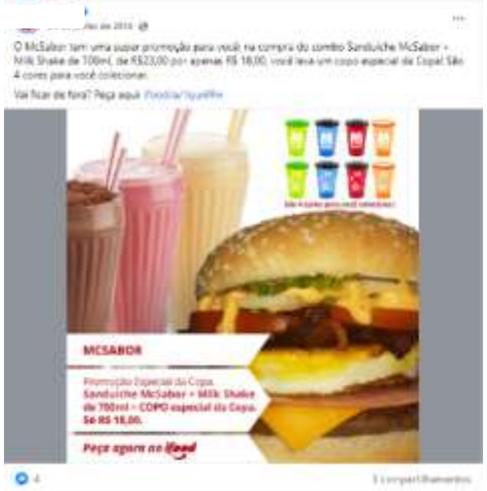

E) Post with brand elements (red color and Ifood logo), interaction with users, through user tagging and engagement (encourage users to tag mothers in the post)

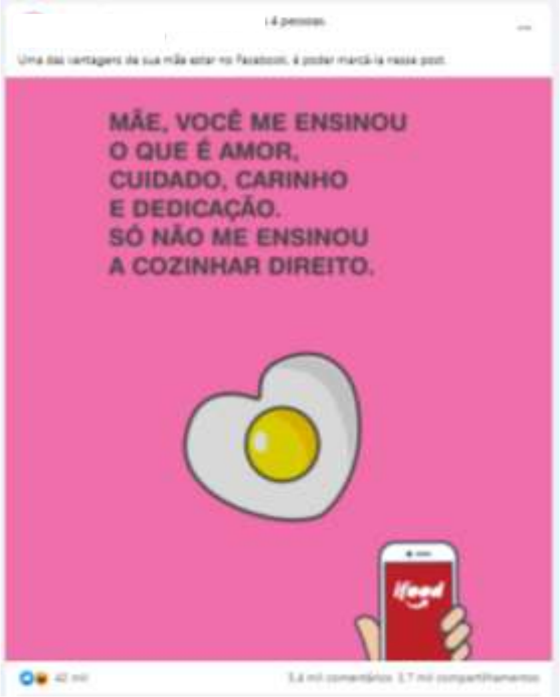

F) User tagging, use of emoticons (symbol of a surprised, happy face and a sandwich) and use of hashtag (#)

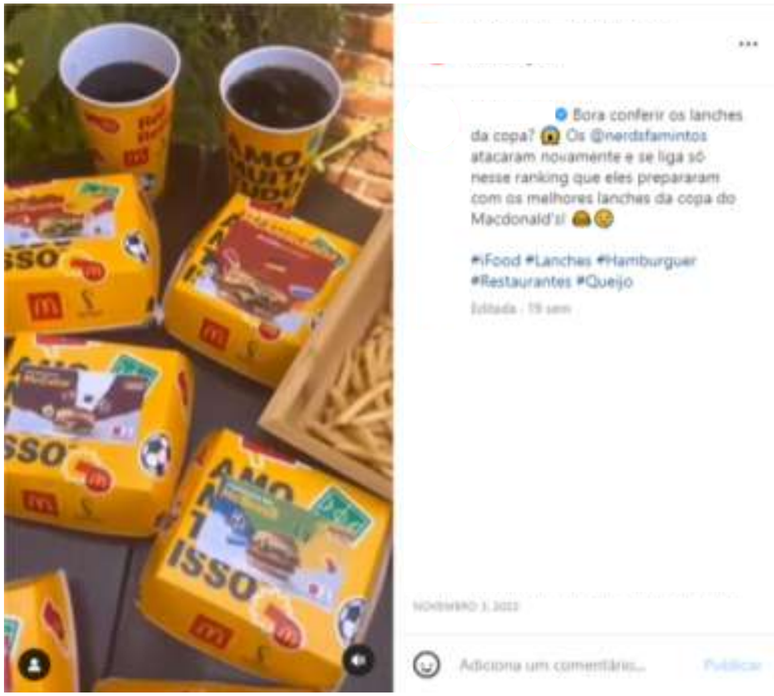

Supplement: Supplementary file 1 [file Data_Sheet_1.PDF]
