# Supplementary material for: Food digital marketing on social media: trends and strategies of Brazil’s leading meal delivery app
Source: Front Nutr. 2025 Jul 1;12:1620348. doi: 10.3389/fnut.2025.1620348 (PMC12265301; doi:10.3389/fnut.2025.1620348)

SUPPLEMENTARY MATERIAL 2 - Examples of content analysis categories present in posts by the main meal delivery app company on Facebook and Instagram, in Brazil 2011-2022.

A) Institutional characteristics of the brand

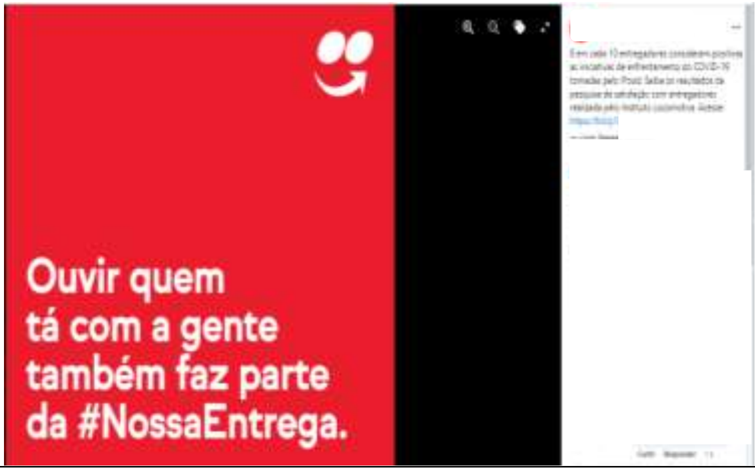

B) Sensory

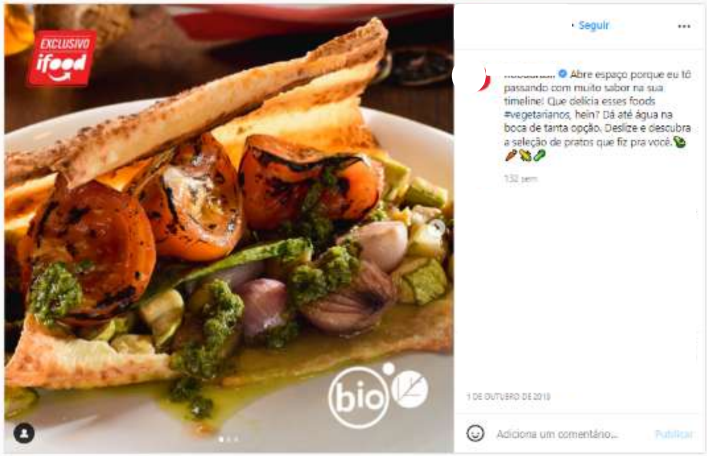

C) Practicality and convenience

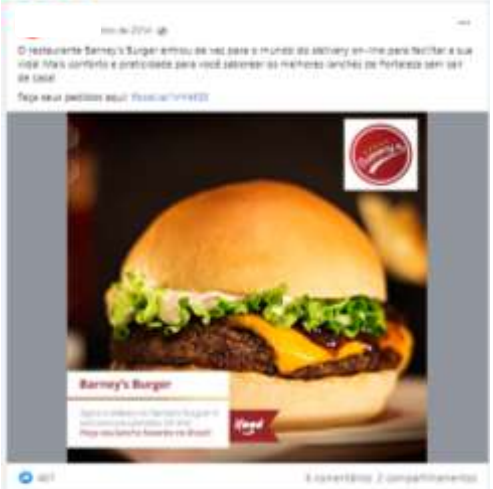

D) Economic benefit

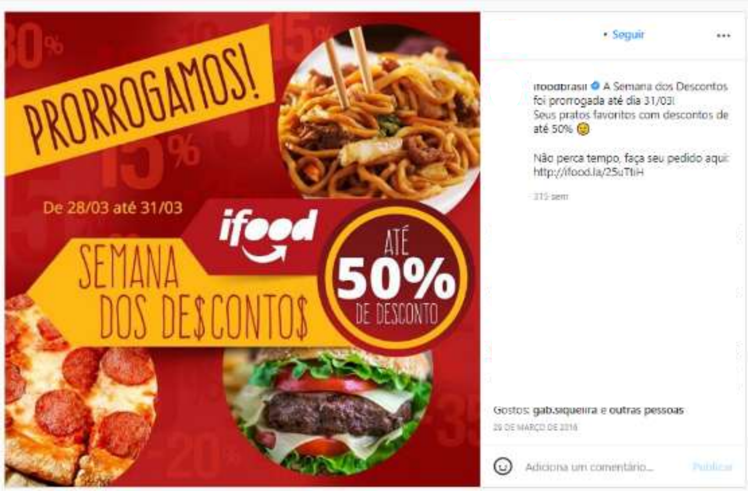

E) Entertainment and social interaction

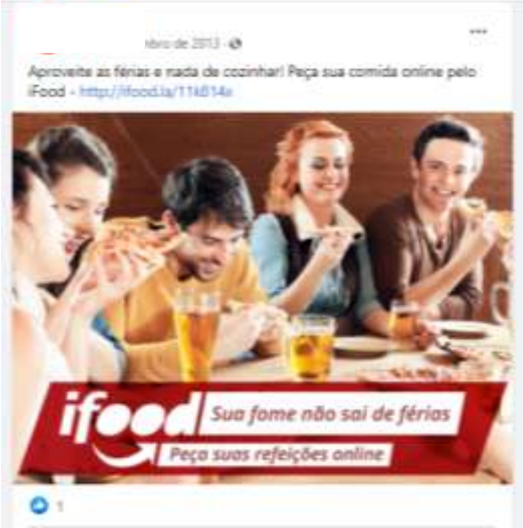

F) Consumption stimulus (in specific situations and contexts)

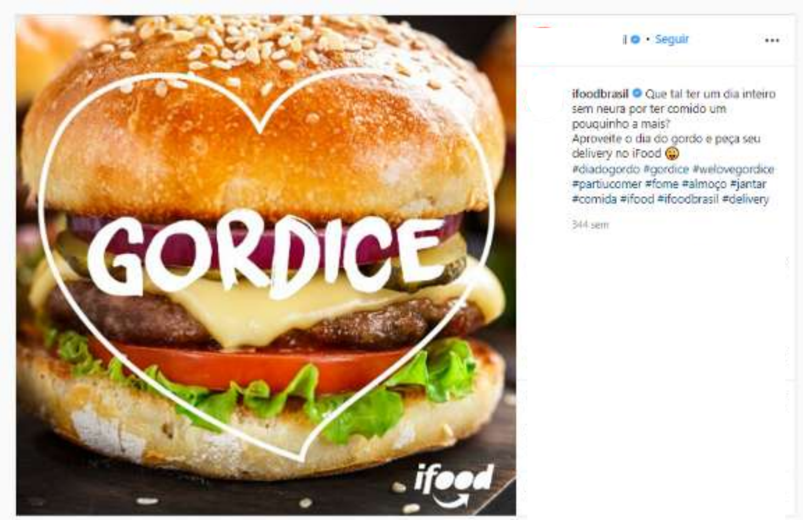

G) Application differentials

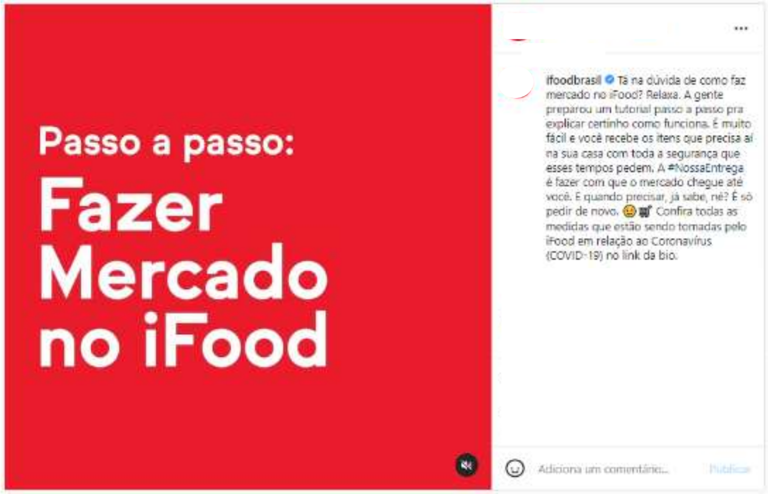

H) Food culture and sophistication

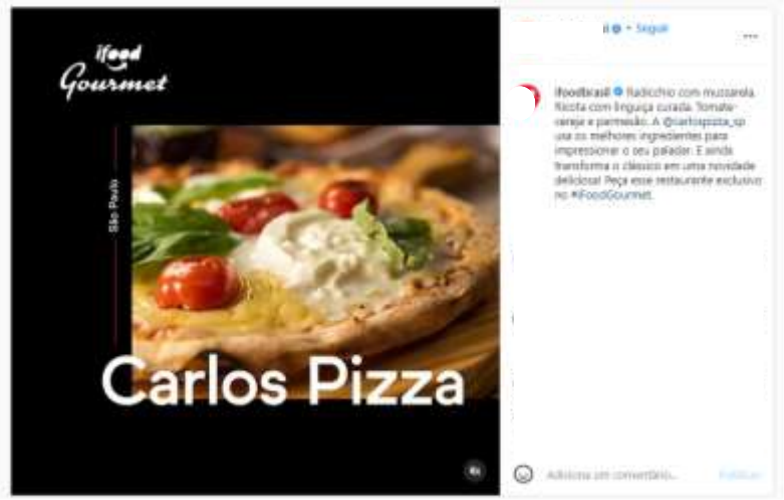

I) Thematic campaigns

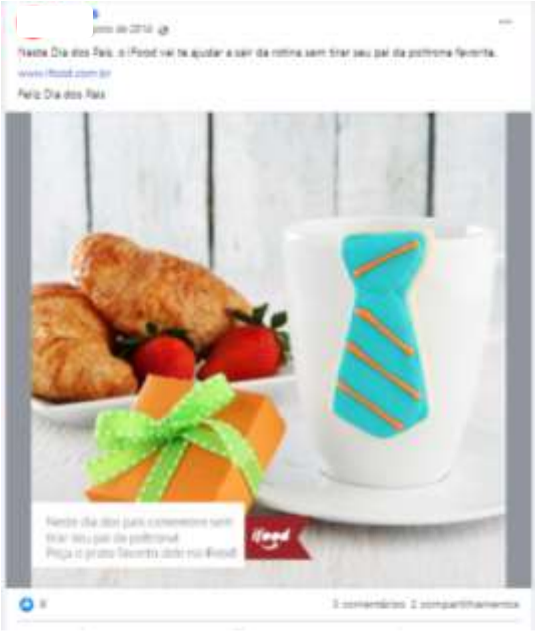

J) Endorsement of public figures

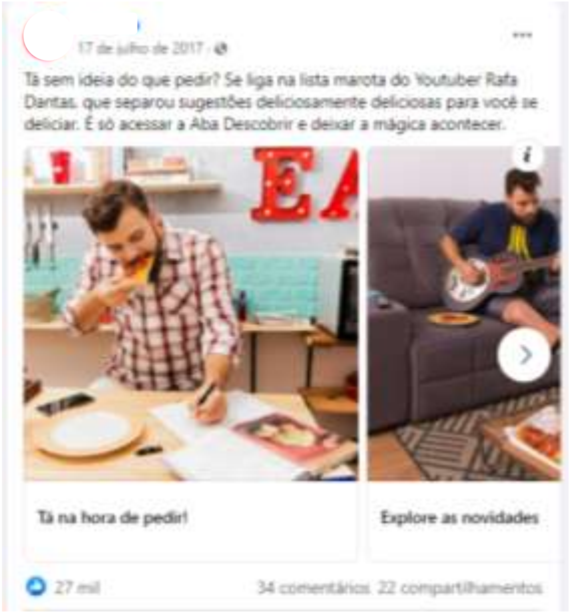

K) Communication and news

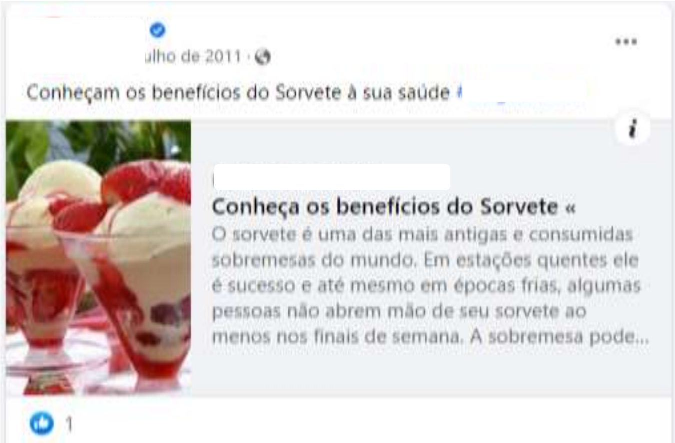

L) Social and corporate responsibility

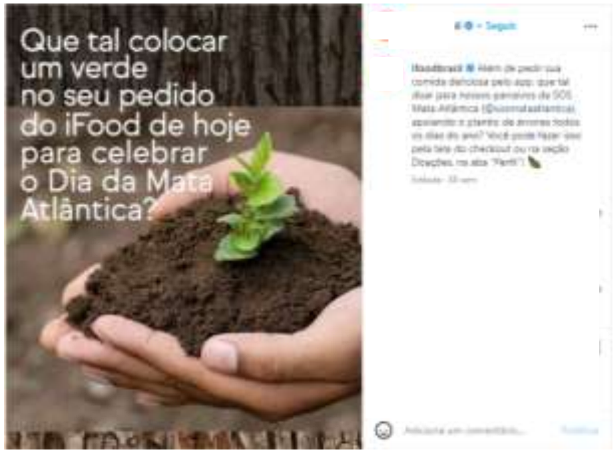

Supplement: Supplementary file 2 [file Data_Sheet_2.PDF]
